# Supplementary material for: Potency Evaluations of Recombinant Botulinum Neurotoxin A1 Mutants Designed to Reduce Toxicity
Source: Int J Mol Sci. 2024 Aug 17;25(16):8955. doi: 10.3390/ijms25168955 (PMC11355004; doi:10.3390/ijms25168955)
Supplement: Supplementary file 1 [file ijms-25-08955-s001.zip › ijms-3073177-supplementary.pdf]

A wild type BoNT/A1

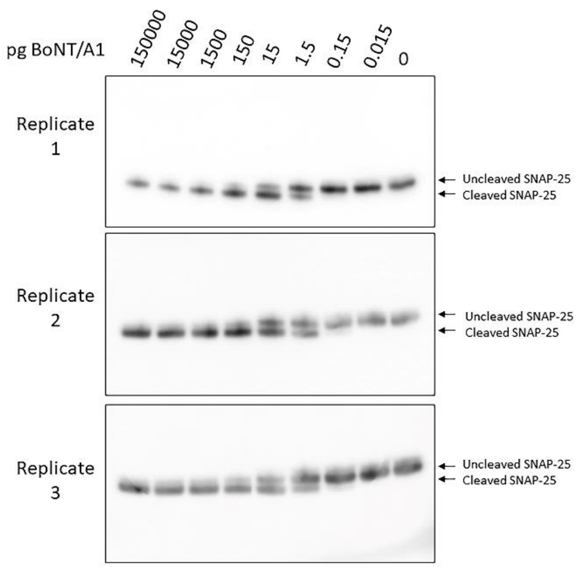

B 7M rBoNT/A1

Repeat Experiment

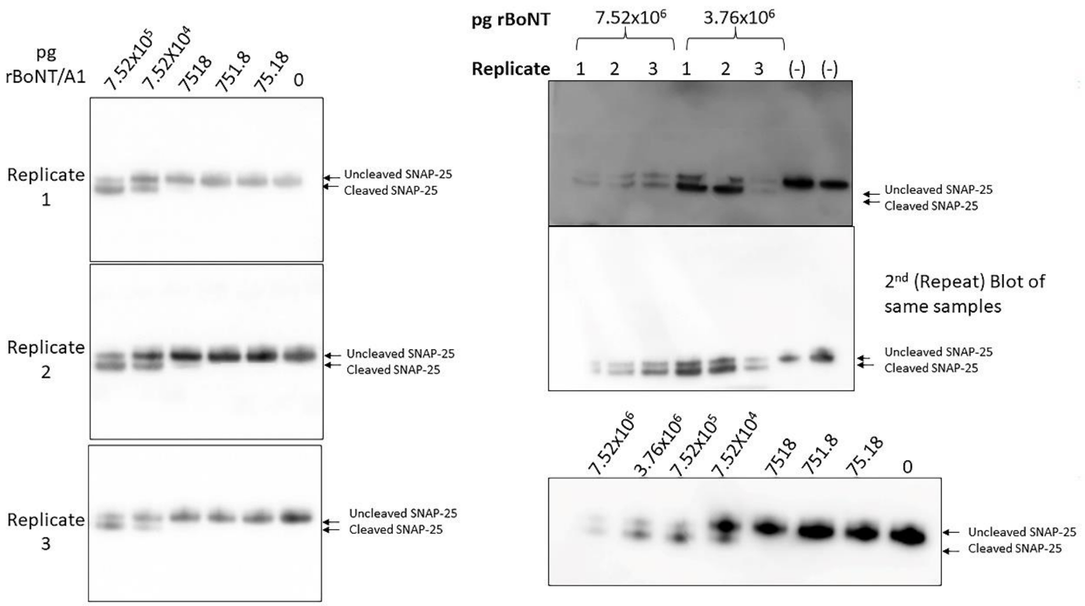

Supplementary Figure S1. The replicates of western blots wt BoNT/A1 and 7M rBoNT/A1 probed with anti-SNAP-25 antibody. A) Cleaved and uncleaved SNAP-25 detected in RSC cell lysates exposed to wt BoNT/A1 for 48 hours. B) Cleaved and uncleaved SNAP-25 detected in RSC cell lysates exposed to 7M rBoNT/A1 for 48 hours. The two highest dilutions were repeated and are shown on a separate blot. The blotting of these

samples was also repeated due to an artifact that may have blocked transfer. Another, separate representative blot is then shown with all dilutions present.

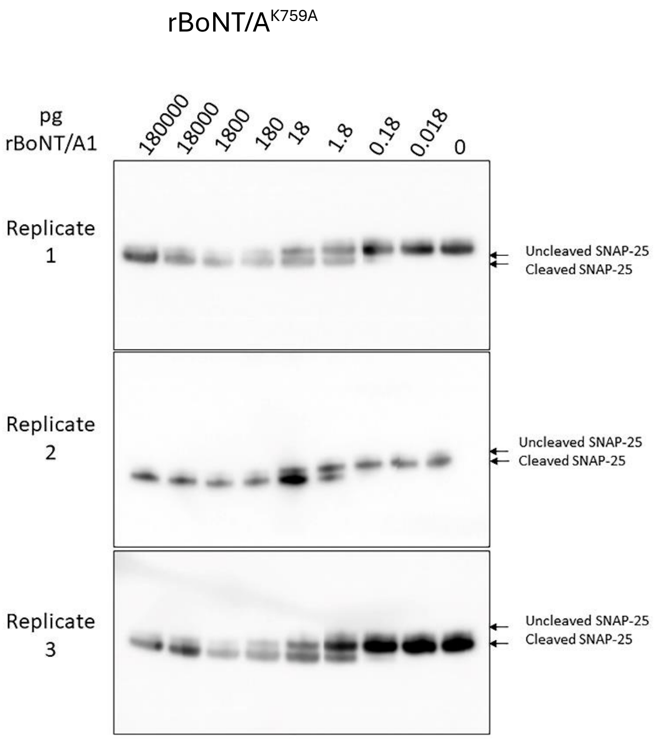

Supplementary Figure S2: The replicates of western blots rBoNT/A1^{K759A} probed with anti-SNAP-25 antibody. Cleaved and uncleaved SNAP-25 detected in RSC cell lysates exposed to rBoNT/A1^{K759A} for 48 hours.

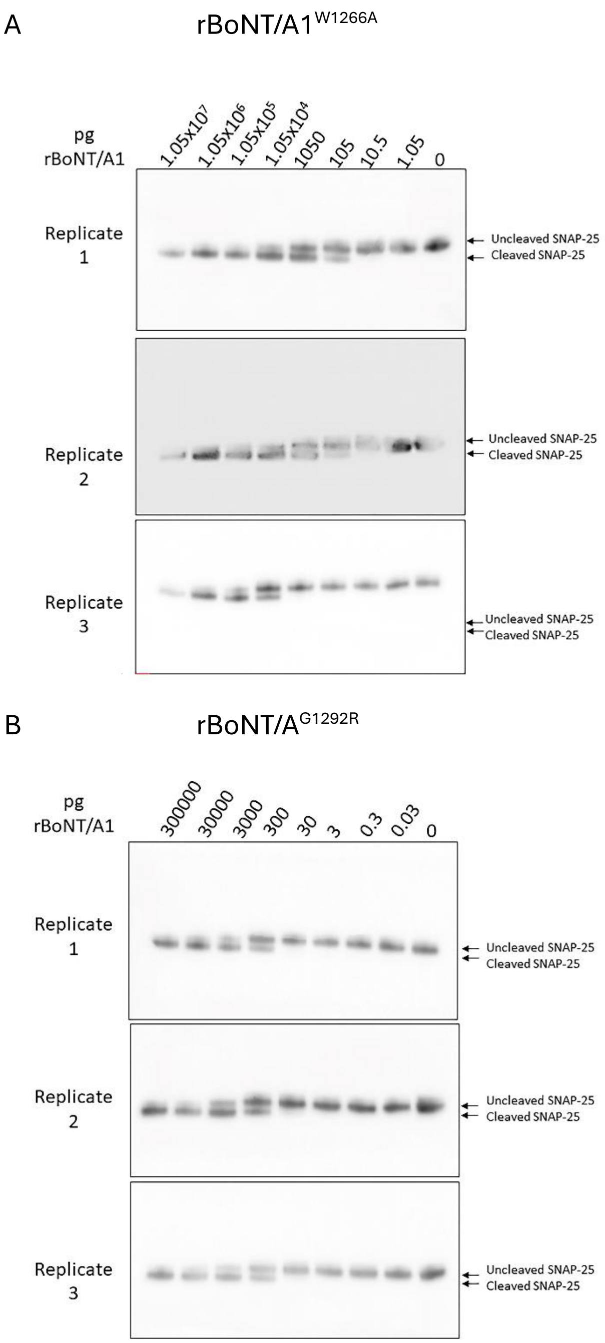

Supplementary Figure S3: The replicates of western blots rBoNT/A1^{W1266A} and rBoNT/A1^{G1292R} probed with anti-SNAP-25 antibody. A) Cleaved and uncleaved SNAP-25 detected in RSC cell lysates exposed to

rBoNT/A1^{W1266A} for 48 hours. B) Cleaved and uncleaved SNAP-25 detected in RSC cell lysates exposed to rBoNT/A1^{G1292R} for 48 hours.

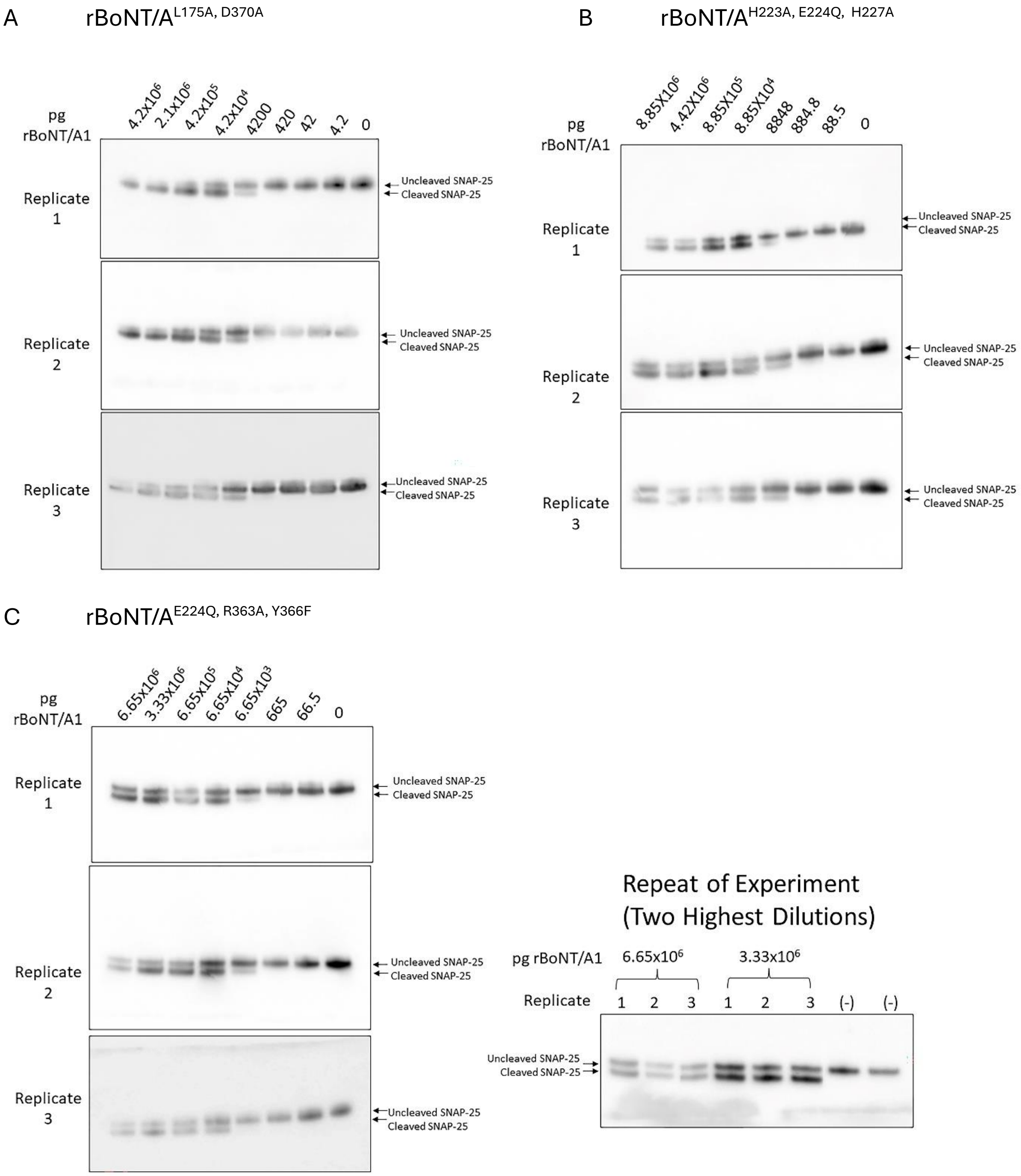

Supplementary Figure S4: The replicates of western blots rBoNT/A1$^{L175A, D370A}$, rBoNT/A1$^{H223A, E224Q, H227A}$ and rBoNT/A1^{E224Q,R363A,Y366F} probed with anti-SNAP-25 antibody. A) Cleaved and uncleaved SNAP-25 detected in

RSC cell lysates exposed to rBoNT/A1$^{L175A, D370A}$ for 48 hours. B) Cleaved and uncleaved SNAP-25 detected in RSC cell lysates exposed to rBoNT/A1$^{H223A, E224Q, H227A}$ for 48 hours. C) Cleaved and uncleaved SNAP-25 detected in RSC cell lysates exposed to rBoNT/A1^{E224Q,R363A,Y366F} for 48 hours. An additional assay of the two highest dilutions was repeated to confirm the previous results and is shown on a separate blot.
